# Financial balance of home nursing care providers in the Czech Republic

**DOI:** 10.1186/s13561-021-00331-1

**Published:** 2021-08-18

**Authors:** Petr Fiala, Iva Holmerova, Tomas Ruda, Michal Steffl

**Affiliations:** 1 Faculty of Humanities, Centre of Expertise Longevity and Long Term Care and Centre of Gerontology, Charles University, Prague, Czech Republic; 2 Faculty of Physical Education and Sport, Charles University, Prague, Czech Republic

**Keywords:** Direct costs, Indirect costs, Long-term care, Home care services

## Abstract

**Background:**

To enable people to live in old age in their own homes often requires specialised home care services. Despite the high importance of these services, the finance of home nursing care (HNC) is still under-investigated in many countries. The aim of this paper was to describe the finance of HNC in the Czech Republic.

**Methods:**

Balancing of revenues and costs was done using structured questionnaires from the closed accounting year 2018 as a monthly average. Nonparametric Kruskal-Wallis and Wilcoxon signed ranks tests were used to test hypotheses.

**Results:**

Data from 62 providers were analysed. The data included information from a total of 2297 patients and 995 employees. The average of total costs were € 17,591.7 (95% CI 14,175.3 - 21,008.1) and average of total revenues were € 17,276.5 (95% CI 13,923.5 - 20,629.5). The average cost per a patient was € 516.0 (95% CI 465.9–566.1) and the average revenues were € 500.1 (95% CI 457.0–543.3).

**Conclusions:**

The overall financial balance of HNC providers seems to be balanced in the Czech Republic. Nevertheless, insurance, although it should, did not cover all the costs. Micro- providers tended to be cheaper regarding the hours worked by nurses.

## Introduction

It is an indisputable fact that the world’s population is ageing. This trend mainly concerns developed countries. Globally, it was estimated that one in six people in the world will be over 65 (16%) by 2050, and in Europe and North America, it could be even one in four people older 65 in 2050. It was also estimated that the number of people aged 80 and over would triple, from 143 million in 2019 to 426 million in 2050 [1]. Alongside the global population ageing, issues related to old age are gaining importance, and several previously less significant problems become of higher importance. One of them is the demand for high-quality HNC services. It is evident and understandable that many old people desire to live in their home until they die [2]. Therefore, long-term home care as a spectrum of services for persons who need assistance in activities of daily living (ADL) has developed into an important topic worldwide. Long-term care (LTC), including HNC, became an important political issue and an essential component of national economies [3]. HNC is an alternative to hospitalisation that could decrease both the demand for hospitalisation as well as length-of-stay. HNC may reduce the costs and decrease risks of complication related to the hospital environment [4]. Governments are concerned about their future sustainability and sufficient resources. In many countries, LTC financing, of which HNC is a part, is a complicated often multi-source process, fragmented among different departments, ministries and levels of state administration or state, regional and municipal governments [5]. In the Czech Republic, people, thanks to the special insurance system, are entitled to free healthcare. This is possible because there is obligation to pay health insurance during the whole working age. According to law, people are not allowed to retire and get the pension until they complete 35 years of paying the insurance. The insurance fee was in 2018 calculated based on the super-gross salary (= gross salary of the employee + social and health insurance contributions paid by the employer). The unified tax rate was set at 45% for all natural persons and it consisted of health insurance (13.5%) and social security (31.5%) [6]. The government then used part of the social security to cover health insurance for children, students, registered unemployed, pensioners, and disabled people. HNC is provided under an Act of Ministry of Health [7]. According to the Health Statistic Office Annual report [8], there were 141 thousand patients in-home care (118 thousand of them 65+) in 2018 out of 10 million people of the overall population. For example, the average of daily treated inpatients was 55 thousand people in medical facilities. That means there were almost three times as many patients in the home care in 2018. The report also declared that the number of patients under HNC had remained stable since 2007. However, the population structure is changing, and numbers of older persons are rapidly increasing, which means that the relative number of older home care patients (per all patients) increases [8, 9]. In 2018, there were in total 6,445,616 visits provided by HNC, meaning that the average number of visits per patient was about 47 [8, 9]. Skilled nurses of several different specialities as general, psychiatric, nutritional, palliative, and so on may serve in HNC in the Czech Republic. However, general care nurses account for the majority of nurses in HNC (84.2%) [10]. The expenditure for HNC from the public health insurance funds was € 82,5 million according to the current data of the Analytical Commission for the Conciliation Procedure in 2018 [11]. Nevertheless, this amount was only a part of all the expenditures from public resources on this area because it did not include other possible sources such as grants or subsidies. Moreover, this amount did not include out-of-pocket payments of individual patients that may not be negligible in some cases. Non-profit organisations and non-governmental private health facilities usually provide HNC in the Czech Republic. A major role among them plays various regional facilities of the Catholic Charities or Evangelical Diaconate; they are even the dominant providers of HNC in some districts [10]. However, it is necessary to mention that some HNC providers are outsourced to agencies in social care establishments; therefore, their costs are not charged to public health insurance costs. Unfortunately, the overall situation about HNC financing is poorly transparent and confusing in the Czech Republic. The system of integrated LTC does not exist (despite a lot of effort to improve the legislation in this field) neither does the LTC insurance. Therefore, the HNC funding remains to be reimbursed from the general health insurance. These embarrassments lead to the supply of insufficiently developed LTC in the home environment, and available services do not meet the needs of older persons with dependency in ADL. In fact, sufficient and fair funding represent a necessary base for service development. Nevertheless, there are not generally available data from official sources. Therefore, the aim of this study was to describe the financial situation in HNC and to estimate financial flows in HNC providers.

## Methods

### Participating providers

The survey was created by balancing of revenues and costs. Cost and revenue items were collected using structured questionnaires from the closed accounting year 2018 as a monthly average from the primary accounting documents of the entities concerned for the “profit and loss account” (or the “income statement”). In total, the 481 home nursing care providers were included in the official health statistics of the Ministry of Health in 2018. They were contacted during the conciliation procedure (regular meetings of care providers). Data collection began in May 2019 and ended in September 2019. Seventy-six home care providers agreed to participate in the survey, from which 62 establishments delivered the relevant data by the end of the data collection procedure. Therefore, the response rate to the section on participating providers was 12.9%; that is relatively a small number that should be mentioned as a limitation of the study.

### Data synthesis

The basic analytical chart of accounts was uniform for all the providers. At this point, it should be noted that synthetic accounts (usually referred to as “accounting”) are regulated in the Czech Republic by the law [12], while analytical (sub-ledger) accounts are not regulated, and it is therefore left to the sole discretion of individual accounting entities. For this reason, it was necessary to choose a uniform structure and procedure for all the participated providers. This approach allowed us distinguishing different types of costs and revenues compatible with generally adopted systems of statistics. The structure of costs is presented in Table 1. Revenues were further methodically divided into payments from health insurance companies and additional items such as out-of-pocket payments from patients, subsidies, grants, sponsor gifts etc. (Table 2). Except for financial information, we collected the total number of staff, number of nurses, and amount of full-time equivalents (FTE) for nurses, total nurses’ working hours a month, and the total number of patients.

**Table 1:** Itemized costs in a month

No.	Item
**I.**	**INDIRECT COSTS**
1.	Leases - offices, warehouses, etc.
2.	Overheads – electricity, heat, cleaning, etc.
3.	Office supplies, costs of telecommunication charges, etc.
4.	Occupational clothing for staff, personal protective equipment
5.	Transport – public transport fares, car fuel, car insurance, etc.
6.	Financial supplies – other insurance, meal vouchers, etc.
7.	Repairs, maintenance, depreciation
8.	Training, education, specialized literature
9.	Accounting, economic and legal services
10.	Other overhead costs – to be specified
11.	Staff costs, including administration and management costs
	**TOTAL INDIRECT COSTS**
**II.**	**DIRECT COSTS**
1.	Staff costs – gross wages, payments of social security and health insurance contributions, overtime work
2.	Medical consumables
3.	Other costs of direct care – to be specified
	**TOTAL DIRECT COSTS**
**III.**	**TOTAL COSTS**

**Table 2:** Itemized revenues in a month

No.	Item
1.	Health insurance revenues
2.	Out-of-pocket payments
3.	Subsidies, grants, etc.
4.	Sponsor gifts, other income
**IV.**	**TOTAL REVENUES**

### Data analysis

In the first step, we divided providers according to the number of staff into three categories - micro-provider < 10, medium-provider < 30, and larger-provider > 30. After, we calculated basic descriptive statistics for the entire sample and each category separately. After that, we calculated averages of balance sheets for the entire sample and each category separately as well. Finally, we calculated averages of selected variables per patient and per hour of the nurse’s work. Differences in those variables among the categories were tested by the nonparametric Kruskal-Wallis test. Within-group difference between costs and revenues were tested by nonparametric paired Wilcoxon signed ranks test. For both tests, the level of significance was set on α = 0.05. We calculated 95% confidence interval (CI) for each variable. To briefly explain how it works, if 95% CI crosses 0 in the cash flow, it means the financial loss or earning was not statistically significant at significance level α = 0.05. All the statistics were carried out in statistical software IBM SPSS Statistics 24.

## Results

Data from 62 providers were analysed; 30 were micro, 24 medium and 8 larger providers. The data included information from a total of 2297 patients and 995 employees. There were 713 nurses out of all the employees. A vast majority of nurses worked on a part-time basis. The average number of all the employees per provider was 16.1 (95% CI 12.5–19.6); for nurses, it was 11.5 (95% CI 9.1–19.9). The average FTE for nurses was 7.7 (95% CI 6.3–9.2). Nurses worked on the average 1824.3 (95% CI 1510.4 - 2198.2) hours a month. All the personnel data logically increased with the size of the provider (Table 3).

**Table 3:** Descriptive statistics of HNC providers included into study

	All	Micro-provider	Medium-provider	Larger-provider
	***N*** = 62	n = 30	n = 24	***n*** = 8
Patients (*N* = 2297)	37.1 (29.3–44.9)	22.7 (16.5–28.8)	39.5 (27.3–51.6)	84.0 (57.8–110.2)
Employees (*N* = 995)	16.1 (12.5–19.6)	7.0 (6.2–7.8)	16.9 (14.6–19.2)	47.3 (37.2–57.3)
Nurses (*N* = 713)	11.5 (9.1–13.9)	6.1 (5.1–7.1)	11.8 (9.8–13.9)	30.6 (20.4–40.8)
FTE for nurses	7.7 (6.3–9.2)	4.9 (3.9–5.9)	8.2 (6.5–10.0)	16.9 (10.5–23.3)
Nurses’ working hours (monthly)	1824.3 (1510.4-2198.2)	1168.8 (932.3–1405.3)	1977.9 (1554.5-2401.3)	4053.9 (2508.4-5599.4)

*Note*: Data are presented as a mean (95% CI); FTE - full-time equivalent

The average of total costs were € 17,591.7 (95% CI 14,175.3 - 21,008.1). The direct costs € 12,871.6 (95% CI 10,239.8 - 15,503.4) were higher than indirect costs € 4656.2 (95% CI 3775.4 - 5537.1). This trend was apparent among all the providers. However, whereas the direct costs were three times higher than indirect costs in the larger-provider category, it was only twice bigger in the micro-providers. The average of total revenues were € 17,276.5 (95% CI 13,923.5 - 20,629.5) and the majority was created by the health insurance revenues € 15,895.7 (95% CI 12,692.6 - 19,098.7). However, the health insurance revenues would not be enough to cover all the costs, because the average insurance revenues to total costs difference were € − 1696.0 (95% CI -2356.1 - -1036.0), which was statistically significant. The overall economy showed a negative trend with the average of cash flow was € -315.5 (95% CI -730.8 - 100.4). However, this trend was not statistically significant in all the providers together as well as in each group separately. The averages of balance sheets are presented in Table 4.

**Table 4:** Balance sheets of providers according to the number of staff

	All	Micro-provider	Medium-provider	Larger-provider
	N = 62	***n*** = 30	***n*** = 24	***n*** = 8
**Total costs**	17,591.7 (14,175.3-21,008.1)	10,718.0 (8200.0-13,236.0)	19,268.5 (14,508.3-24,028.7)	38,337.8 (24,673.5-52,002.1)
Indirect costs	4656.2 (3775.4-5537.1)	2938.7 (2108.2-3769.1)	5282.4 (3915.9-6648.8)	9218.5 (6302.7-12,134.3)
Direct costs	12,871.6 (10,239.8–15.503.4)	7905.8 (6074.7-9737.0)	13,662.9 (10,096.8-17,229.0)	29,119.3 (17,522.5-40,716.1)
**Total revenues**	17,276.5 (13,923.5-20,629.5)	10,876.7 (8332.8-13,420.6)	18,634.1 (13,805.6-23,462.5)	37,203.2 (23,681.6-50,724.8)
Health insurance revenues	15,895.7 (12,692.6-19,098.7)*	10,103.3 (7599.7-12,606.8)*	17,100.0 (12,296.9-21,903.0)*	34,004.4 (20,748.6-47,260.2)*
Out-of-pocket payments	86.2 (−4.1 (176.5)	45.5 (−8.3–99.4)	128.4 (−99.4–356.2)	112.3 (−67.1–291.6)
Subsidies, grants, etc.	539.0 (318.4–759.5)	216.6 (−0.7–433.9)	573.0 (259.7–886.3)	1645.5 (618.0–2673.1)
Sponsorship gifts, other income	711.9 (506.4–917.4)	458.4 (252.5–664.4)	785.7 (506.7–1064.7)	1441.1 (227.1–2655.1)
Insurance revenues to total costs difference	-1696.0 (− 2356.1--1036.0)	− 614.7 (− 1427.8–228.4)	− 2168.6 (− 2981.3--1355.9)	− 4333.4 (− 7212.1--1454.7)
**Cash flow**	−315.2 (− 730.8–100.4)	158.7 (− 409.3–726.7)	−634.5 (− 1285.5–16.5)	− 1134.5 (− 2778.5–509.4)

*Note*: Data are presented as a mean (95% CI); *statistically significant difference between the total cost and health insurance revenues

The average cost per a patient was € 516.0 (95% CI 465.9–566.1) and the average revenues were € 500.1 (95% CI 457.0–543.3). There was a significant negative cash flow when the total costs were deducted from the total insurance revenues € -64.3 (95% CI -97.6 - -31.0). Similar statistically significant negative cash flow was also in all the groups. Negative cash flow was also found after deducting the total revenues from total costs. However, these negative cash flows were not statistically significant. The averages of all these variables differed across the groups but not significantly. The averages cash flow per patient is presented in Table 5.

**Table 5:** Costs and revenues per patients depending on the size of provider

	All	Micro-provider	Medium-provider	Larger-provider	
	N = 62	n = 30	n = 24	n = 8	p value
Total costs	516.0 (465.9–566.1)	505.0 (432.6–577.5)	550.2 (454.3–646.2)	454.5 (397.8–511.3)	0.569
Total revenues	500.1 (457.0–543.3)	497.8 (442.7–552.9)	522.4 (432.0–612.7)	442.2 (381.6–502.8)	0.469
Total costs to revenues difference	−15.9 (−36.8–5.0)	−7.3 (− 42.6–28.1)	−27.8 (−61.3–5.49)	−12.3 (− 31.8–7.2)	0.648
Insurance revenues to total costs difference	−64.3 (−97.6--31.0)	−52.5 (−113.4--8.5)	− 81.8 (− 125.9--37.7)	−56.2 (−92.4--20.1)	0.313

*Note*: Data are presented as a mean (95% CI); *p* values were calculated by Kruskal-Wallis test

If we calculated all the costs and revenues based on a nurse’s working hour, the total cash flow was negative. The total cost per hour was € 9.2 (95% CI 8.8–9.6) and total revenues € 9.1 (95% CI 8.6–9.5). With the size of the provider the negative difference increased, however, the differences were not statistically significant in any cases. Nevertheless, differences between the total costs and payments from health insurance companies were statistically significant in all the cases and the difference increased with the size of the provider (Table 6).

**Table 6:** Costs and revenues per an hour of the nurse’s work depending on the size of provider

	All	Micro-provider	Medium-provider	Larger-provider	
	N = 62	***n*** = 30	***n*** = 24	***n*** = 8	p value
Indirect costs	2.5 (2.3–2.7)	2.4 (2.0–2.7)	2.7 (2.3–3.0)	2.5 (1.7–3.3)	0.401
Direct costs	6.7 (6.3–7.0)	6.5 (6.0–7.1)	6.7 (6.2–7.1)	7.1 (6.7–7.6)	0.465
Total costs	9.2 (8.8–9.6)	8.8 (8.1–9.5)	9.5 (8.9–10.1)	9.7 (8.7–10.6)	0.217
Total revenues	9.1 (8.6–9.5)	9.0 (8.1–9.9)	9.0 (8.5–9.6)	9.4 (8.3–10.5)	0.825
Health insurance revenues	8.3 (7.8–8.8)*	8.3 (7.4–9.3)*	8.2 (7.5–8.9)*	8.5 (7.4–9.6)*	0.913

*Note*: Data are presented as a mean (95% CI); p values were calculated by Kruskal-Wallis test; *statistically significant difference between the total cost and health insurance revenues

## Discussion

This study can be seen as a first attempt to analyse the HNC financing on the microeconomic level. At this point, it should be mentioned that the overall financial balance of HNC providers was not well balanced. Even though a majority of HNC was reimbursed through general health insurance, providers needed to obtain additional resources to achieve financial balances. As it was mentioned above, mainly non-profit organisations provide HNC in the Czech Republic. A long-term unbalanced financial situation can lead these organisations into debt [13].

Comparing costs for HNC among different countries is a quite complicated process that might not make sense because economic power differs across countries, which may skew the comparison. For example, the diversity of rural areas in terms of functions, economic development level and access to social and technical infrastructure [14] and the overall availability of health care in the region may play a role in case of cost of healthcare [15]. Financial measures are not interchangeable and accounting constructs are multidimensional [16]. Extra-organizational factors that affect the economy and the market including corporate earnings, tax rates, stock and bond returns, and fluctuations in government funding theoretically influence the estimation [17]. However, the average cost of formal care per a patient € 516 in this study was very low in comparison to Norway study where the formal cost per a patient was estimate at € 1820 [18], or to German study at € 1136 [19] or to Taiwanese study at 4188 US dollars [20]. The costs for HNC were relatively low, perhaps due to nurses working mostly on a part-time basis. It is not entirely clear why this is happening and whether the costs would not increase if the nurses worked full time. Nurses’ salaries range from € 1070 for younger nurses with less experience to € 1842 for nurses with more than 30 years of practice. However, most nurses were below the average salary of € 1416 in the Czech Republic [21]. It may be another reason why HNC stays relatively cheap in the Czech Republic compared to other countries.

Another important question is whether HNC is more economical or effective than institutional care. According to some studies, the HNC had potential on reduction in public expenditure, and according to some others, HNC did not have that effect [18, 22]. However, much more important and sensible is the information that HNC can also save on care costs in addition to patient benefits. For example, home care provision had been demonstrated to be more effective and efficient than institutionalised care [23–25], home visits implemented by a multidisciplinary team appeared to help slow down the health-related quality of life among older adults [26] without accruing additional costs [27], and enhanced quality of life while not increasing the overall costs of health care [25]. In-home palliative team care was cost-effective; it increased the chance of dying at home by 10%, increased the average number of days at home (6 days) and quality-adjusted life-days (0.5 days), and it reduced costs by approximately $4400 per patient [28]. Community-based specialist palliative care was associated with hospital cost reductions across multiple life-limiting conditions [29]. However, HNC is one of means that enable people to live in old age in their own homes even if they are partly dependent in ADL. HNC may offer clinical benefits across a number of important health dimensions and may be socially desirable alternative to institutionalisation [30].

Despite high importance, only a little piece of information is available regarding its financing from public resources in many countries [31]. Because of a relatively well prepared social system a covering of all the health-related expenses by health insurance is assumed in the Czech Republic. Unfortunately, as our results showed, health insurance revenues hardly cover all the costs required for non-governmental, non-profit facilities providing HNC. However, our results confirmed the previous Holmerova and Prochazka [32] findings. These authors published a paper about a non-profit organisation that provided HNC to older adults. Their calculation proved that one hour of HNC’s cost was € 13.6 whereas revenues were only € 10.1, which made a deficit of € 3.5 per hour. In this case, that deficit was refunded by the local authority to provide sufficient and adequate quality home-care for older persons in the community [32]. Nevertheless, this study as well as ours study focused only on the formal care costs. However, informal care comprised the main part of the total cost of care [33, 34]. The proportion of informal care was almost 88% of the total cost in older adults with dementia according to Holmerova et al. [34]. Therefore, it must be mentioned that our estimation does not by far cover all the expenditure associated with homecare in the Czech Republic.

## Conclusions

In conclusion, the overall financial balance of HNC providers seems to be balanced in the Czech Republic. Nevertheless, that happened mostly because of additional financial resources. Insurance, although it should, did not cover all the costs. The size of the provider did not play a crucial role. Nevertheless, for micro- providers, it tended to be cheaper regarding the hours worked by nurses. Price assessment or costs comparison between institutional and homecare was not possible because relevant data from institutional care were not available. In the future, therefore, extensive investigations should be carried out into both HNC and institutional care costs.

## Data Availability

Request for data sets generated and analysed during the current study can be addressed to the corresponding author.

## Abbreviations

ADL: activities of daily living
CI: confidence interval
€: Euro
FTE: full-time equivalents
HNC: home nursing care
LTC: long-term care
